# Chloroquine Suppresses Effector B-Cell Functions and Has Differential Impact on Regulatory B-Cell Subsets

**DOI:** 10.3389/fimmu.2022.818704

**Published:** 2022-02-08

**Authors:** Xin Ma, Yang Dai, Oliver Witzke, Shilei Xu, Monika Lindemann, Andreas Kribben, Sebastian Dolff, Benjamin Wilde

**Affiliations:** ^1^ Department of Nephrology, University Hospital Essen, University of Duisburg-Essen, Essen, Germany; ^2^ Department of Nephrology, First Affiliated Hospital of Chengdu Medical College, Chengdu, China; ^3^ Department of Infectious Diseases, West German Centre of Infectious Diseases, University Hospital Essen, University of Duisburg-Essen, Essen, Germany; ^4^ Institute for Transfusion Medicine, University Hospital Essen, University of Duisburg-Essen, Essen, Germany

**Keywords:** chloroquine, regulatory B (Breg) cells, renal transplantation, B-cells, effector B-cells

## Abstract

**Objectives:**

Chloroquine (CQ) is approved for treatment of B-cell mediated diseases such as rheumatoid arthritis and systemic lupus erythematosus. However, the exact mode of action in these diseases has not been studied and it remains unclear which effect CQ has on B-cells. Thus, it was the aim of this study to investigate to which extent CQ affects functionality of effector and regulatory B-cell.

**Methods:**

For this purpose, B-cells were isolated from peripheral blood of healthy controls and renal transplant patients. B-cells were stimulated in presence or absence of CQ and Interleukin-10 (IL-10) and Granzyme B (GrB) secretion were assessed. In addition, effector functions such as plasma cell formation, and Immunoglobulin G (IgG) secretion were studied.

**Results:**

CQ suppressed Toll-Like-Receptor (TLR)-9 induced B-cell proliferation in a dose-dependent manner. IL-10^pos^ regulatory B-cells were suppressed by CQ already at low concentrations whereas anti-IgG/IgM-induced GrB secreting regulatory B-cells were less susceptible. Plasma blast formation and IgG secretion was potently suppressed by CQ. Moreover, purified B-cells from renal transplant patients were also susceptible to CQ-induced suppression of effector B-cell functions as observed by diminished IgG secretion.

**Conclusion:**

In conclusion, CQ had a suppressive effect on IL-10 regulatory B-cells whereas GrB secreting regulatory B-cells were less affected. Effector functions of B-cells such as plasma blast formation and IgG secretion were also inhibited by CQ. Effector B-cells derived from renal transplant patients already under immunosuppression could be suppressed by CQ. These findings may partly explain the clinical efficacy of CQ in B-cell mediated autoimmune diseases. The application of CQ in other disease contexts where suppression of effector B-cells could offer a benefit, such as renal transplantation, may hypothetically be advantageous.

## Introduction

Chloroquine and its derivate hydroxychloroquine (CQ) have originally been developed as antimalarial drugs. In addition, CQ was investigated in multiple clinical trials for efficacy in inflammatory diseases such as rheumatoid arthritis (RA) and systemic lupus erythematosus (SLE) (1, 2). In these trials, CQ was shown to reduce disease activity and thus it is now an established therapeutic drug which is used regularly. The mechanisms by which CQ reduces disease activity have not been unraveled completely; experimental evidence indicates that it interferes with lysosomal acidification, intra-cytoplasmatic calcium mobilization, antagonizes nucleic acid ligands for toll-like-receptors, inhibits autophagy and suppresses the cyclic GMP-AMP-synthetase/stimulator of interferon genes (cGAS/STING) pathway (3). Whereas the effects of CQ on T-cells have been studied by several groups, less is known on the impact of CQ on B-cell effector and regulatory function (3, 4). It was the aim of this study to assess the effect of CQ on effector and regulatory B-cell populations.

## Material and Methods

### Patients and Samples

Buffy coats from healthy blood donors provided by the Institute for Transfusion Medicine were used for the experiments. In addition, 22 patients after renal transplantation were enrolled. Details on the patient demographics are given in **Table 1**. This study was approved by the local ethics committee and all patients provided informed consent.

**Table 1:** Patient’s characteristics.

	Renal Transplant Patients (n = 22)
**Age, mean ± SD, years**	58 ± 14
**Sex (female/male)**	5/17
**Time since transplantation (months)**	142 ± 106
**# of patients ≥1 previous RTX**	0
**# of HLA-mismatches (HLA-A,-B,-DR)**	3 ± 2
**Immunosuppressants at the time of sampling (# of patients treated)**	
Tacrolimus	15
Cyclosporine A	4
Mycophenolate	18
mTOR inhibitor	5
Steroids	11
**CMV status at the time of RTX (D/R)**	
+/+	4
+/-	3
-/+	6
-/-	5
unknown	4

### Peripheral Blood Mononuclear Isolation

Peripheral blood mononuclear cells (PBMCs) were isolated by density gradient centrifugation using Lymphoprep (Stemcell, Cologne, Germany). In case purified B-cells were used for the experimental procedures, B-cells were isolated from PBMCs using a bead-/column-based magnetic separation method (B-cell isolation kit II, Miltenyi Biotec, Bergisch Gladbach, Germany). B-cells were purified by negative selection and purity was typically above 90%. In case B-cells were isolated from renal transplant patients, B-cells were directly isolated from whole blood using a magnetic bead-based negative selection system (MACSXpress Whole Blood B cell isolation kit, Miltenyi Biotec) and purity was typically above 90%. To track proliferation, cells were labeled with 2 µM carboxyfluorescein-succinimidyl-ester (CFSE, Sigma Aldrich, Taufkirchen, Germany).

### Cell Culture

PBMCs or isolated B-cells were cultured in RPMI1640 Glutamax (Thermo Fisher Scientific, Darmstadt, Germany) supplemented with 100 U/ml Penicillin (Sigma Aldrich), 100 µg/ml Streptomycin (Sigma Aldrich), 10% fetal calf serum (Greiner-Bio one, Frickenhausen, Germany), NEAA (Sigma Aldrich) and sodium pyruvate (Sigma Aldrich) at a concentration of 0.5x10^6^ cells/ml in 96-well U-bottom plates (Sigma Aldrich). Depending on the assay, cells were cultured for three to six days in a 5% CO2 atmosphere at 37°C. To induce Interleukin (IL)-10 producing Breg, cells were stimulated with CpG (ODN2006, 0.1 μM, Invivogen, Toulouse, France), Poly-S (resiquimod plus IL-2, 1:1000 final dilution, CTL Europe GmbH, Bonn, Germany) or anti-human Immunoglobulin G/M (IgG/IgM, 6 μg/ml and 6.5 μg/ml, Jackson Immunoresearch Europe Ltd, Cambridge, United Kingdom) for 72 hours, followed by restimulation with phorbol 12-myristate-13-acetate (PMA) (10 ng/ml) and ionomycin (1 µg/ml, both Sigma-Aldrich) in presence of brefeldin A (5 ug/ml, BFA, BD Biosciences). For detection of Granzyme B producing (GrB^pos^) B-cells, B-cells were stimulated with stimulated with CpG (ODN2006, 0.1 μM) or anti-human IgG/IgM (6 μg/ml and 6.5 μg/ml, Jackson Immunoresearch Europe Ltd) in presence of IL-21 (50 ng/ml, Miltenyi Biotec) for 72 hours, followed by restimulation with phorbol 12-myristate 13-acetate (PMA, 10 ng/ml, Sigma Aldrich) and ionomycin (1 µg/ml, Sigma-Aldrich) in presence of brefeldin A (BFA, BD Biosciences). For the plasma blast formation assay, cells were stimulated for six days with either CpG (ODN2006, 0.1 μM, Invivogen) or anti-human IgG/IgM (6 μg/ml and 6.5 μg/ml, Jackson Immunoresearch Europe Ltd) in presence of IL-2 (50 ng/ml, Miltenyi Biotech) and IL-21 (50 ng/ml, Miltenyi Biotech). To detect IgG secretion, isolated B-cells or PBMCs were stimulated with Poly-S (resiquimod plus IL-2, 1:1000 final dilution, CTL Europe GmbH) for four days and then transferred to a prepared EliSpot plate to be incubated for further 24 hours. CQ (chloroquine, Cyto-ID kit, Enzo Life Sciences, Lörrach, Germany) was used at different concentrations for the assays. Tacrolimus (1.25 ng/ml, Sigma Aldrich) and rapamycin (12.5 ng/ml, Sigma Aldrich) were used in selected experiments as comparators with a known immunosuppressive effect on B-cells. CNIs are also commonly used in other autoimmune diseases such as systemic lupus erythematosus as well as CQ.

### EliSpot Assay

For detection of IgG-secreting human cells, a commercially available EliSpot kit was used (Human IgG single-color EliSpot, CTL Europe GmbH) according to the manufacturer’s instructions. For the final incubation step, fixed numbers of cells were used (500 or 1000 cells per well) and spots were counted using an EliSpot Reader (AID, Straßberg, Germany).

### Flow Cytometry

For GrB and IL-10 detection, cells were harvested at the end of the culture period and stained with anti-CD19 Pacific Blue (clone J3-119, Beckman Coulter, Krefeld, Germany) and 7AAD (BioLegend, Eching, Deutschland) followed by fixation/permeabilization (Cytofix/Cytoperm kit, BD Biosciences, Heidelberg, Germany). Cells were then stained intracellulary for GrB (anti-GrB, clone GB11, PE, eBioscience) or IL-10 (anti-human IL-10, APC, clone JES3-9D7, Biolegend). For the plasma blast formation assay, cells were harvested after culture and stained with anti-human CD19 (clone J3-119, FITC, Beckman Coulter), anti-human CD38 (clone HIT-2, PE, Biolegend), anti-human CD27 (clone O323, APC-H7, Biolegend). Appropriate isotype controls were used to confirm specificity of staining. Flow cytometric measurement was performed the same day with a fluorescence activated cell sorter (FACS) NAVIOS™ from Beckman Coulter. Kaluza Version 2.1 (Beckman Coulter) was used to analyze FACS data.

### Statistics

All values are expressed as mean ± standard deviation (SD). The significance for the differences between groups was determined by ANOVA and Dunnetts test. Differences were considered statistically significant at a p-value <0.05.

## Results

### Chloroquine Suppresses CpG-Induced B-Cell Proliferation

**Figure 1:**
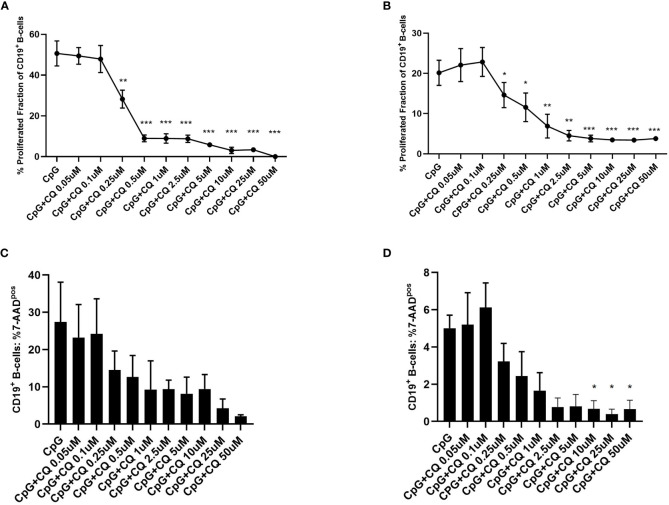
Dose-dependent suppression of TLR9-induced B-cell proliferation by CQ. PBMCs or purified B-cells were labeled with CFSE and stimulated with CpG in presence of different concentrations of CQ. After 72 hours, CD19^+^ B-cell proliferation was determined by CFSE-dilution. **(A)** Impact of CQ on B cell proliferation with CpG stimulation in PBMCs. **(B)** Impact of CQ on B cell proliferation with CpG stimulation in purified B cells. **(C)** Impact of CQ on vitality of B-cells after stimulation with CpG in PBMCs. **(D)** Impact of CQ on vitality of B-cells after stimulation with CpG in purified B-cells, P-values were calculated by repeated-measures ANOVA and correction for multiple comparisons were done by Dunnett’s test. *p < 0.05, **p < 0.01, ***p < 0.0001 (against CpG).

**Figure 2:**
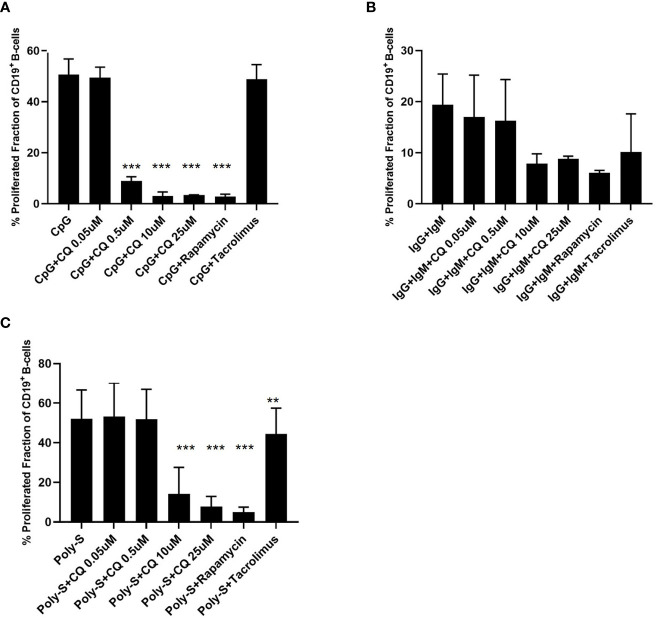
CQ affects B-cell proliferation depending on the stimulus. PBMCs were isolated and labeled with CFSE followed by stimulation with different stimuli in presence of CQ at four concentrations (0.05 µM, 0.5 µM, 10 µM, 25 µM). Rapamycin and tacrolimus were also used as controls. After 72 hours, CD19^+^ B-cell proliferation was determined by CFSE-dilution. **(A)** Impact of CQ on B cell proliferation upon stimulation with CpG. **(B)** Impact of CQ on B cell proliferation upon IgM/IgG stimulation. **(C)** Impact of CQ on B cell proliferation upon Poly-S stimulation. P-values were calculated by repeated-measures ANOVA and correction for multiple comparisons were done by Dunnett’s test. **p < 0.01, ***p < 0.0001 (against CpG, IgG+IgM or Poly-S as control conditions).

To study the effect of CQ on B-cells, purified B-cells or PBMCs were used. First, it was determined whether CQ had impact on the proliferation of stimulated B-cells. For this purpose, CFSE-labeled cells were stimulated with CpG in presence or absence of CQ. CQ suppressed B-cell proliferation in a dose-dependent manner (**Figure 1**). This effect was observed in purified B-cell and PBMCs cultures. Enhanced cell death was not observed after stimulation in presence of CQ (**Figures 1C, D** **)**. Next, it was determined whether the type of stimulus has impact on CQ-mediated suppression. The type of stimulus was chosen according to the expected strengths of stimulation and to the biological context in which B-cell activation occurs. It is generally thought that full B-cell activation requires different signals under physiological circumstances *in vivo (* 5, 6). Binding of the cognate antigen to the B-cell receptor (BCR) may serve as initial signal and promotes antigen processing (5, 6). Additional signals amplifying B-cell activation can be mediated by T-cells or by pathogen-associated molecular patterns (PAMP) being recognized by TLR (7, 8). TLR7 and TLR9 bind different ligands. While TLR7 senses single-stranded RNA (ssRNA), TLR9 senses hypomethylated CpG DNA motifs (9–11). Both ssRNA and CpG are pathogen-associated molecular patterns (PAMP) and recognition of these PAMP is pivotal to establish a sufficient immune response. ssRNA is a PAMP mostly associated with viral pathogens such as influenza whereas CpG motifs as PAMP can be found in bacterial and viral pathogens (9–11). However, there is some evidence that also host-derived ssRNA and CpG acting as damage-associated molecular patterns (DAMP) may activate the respective TLR under specific circumstances (9). As both TLR-pathways have an important role in host defense and may have a role in the pathogenesis of B-cell mediated diseases, TLR7 and TLR9 agonists were used for *in vitro* experiments. Thus, established BCR- and TLR-based *in vitro* activation protocols were chosen for comprehensive characterization of B-cells. B-cells stimulated with CpG were more susceptible to CQ-induced suppression than B-cells stimulated *via* the B-cell receptor (BCR) using anti-IgG/IgM or *via* TLR7/8 using Poly-S, i.e. resiquimod (**Figure 2**). Rapamycin reduced proliferation of B-cells stimulated with CpG or Poly-S whereas Tacrolimus-mediated suppression was minor (**Figures 2A, C** **)**.

### IL-10^pos^ Breg Are More Susceptible to CQ-Mediated Suppression Than Anti-BCR Induced GrB^pos^ Breg

**Figure 3:**
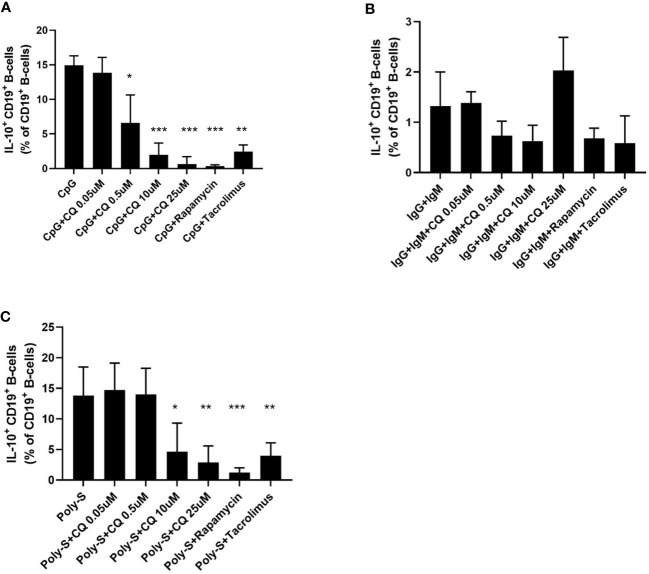
Effect of CQ on IL-10^pos^ regulatory B-cells. PBMCs were cultured in presence of different stimuli and CQ at four concentrations (0.05 µM, 0.5 µM, 10 µM, 25 µM) for 72 hours. Rapamycin and tacrolimus were also used as controls. After 72 hours, the production of IL-10 by CD19^+^ B-cells was determined by flow cytometry. **(A)** Impact of CQ on IL-10^+^ CD19^+^ B-cells upon CpG stimulation in PBMC. **(B)** Impact of CQ on IL-10^+^ CD19^+^ B-cells upon IgG+IgM stimulation in PBMCs. **(C)** Impact of CQ on IL-10^+^ CD19^+^ B-cells upon Poly-S stimulation in PBMCs. P-values were calculated by repeated-measures ANOVA and correction for multiple comparisons were done by Dunnett’s test. *p < 0.05, **p < 0.001, ***p < 0.0001 (against CpG, IgG+IgM or Poly-S as control conditions).

**Figure 4:**
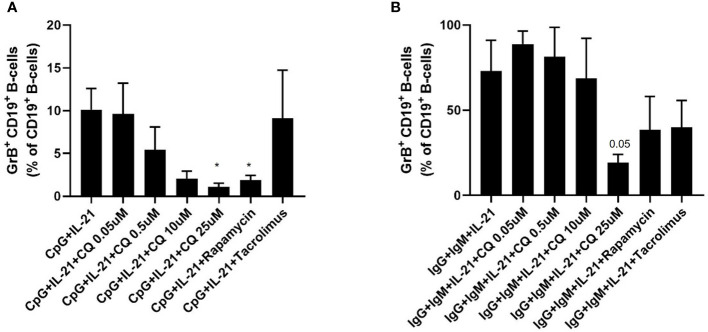
Effect of CQ on GrB^pos^ regulatory B-cells. Purified B cells were isolated and stimulated *via* TLR9 or BCR plus IL-21, in presence or absence of CQ, rapamycin or tacrolimus for 72 hours. After 72 hours, GrB^+^ CD19^+^ B-cells were determined by flow cytometry. **(A)** Impact of CQ on GrB^+^ CD19^+^ B-cells upon CpG plus IL-21 stimulation. **(B)** Impact of CQ on GrB^+^ CD19^+^ B-cells upon IgG/IgM plus IL-21 stimulation. P-values were calculated by repeated-measures ANOVA and correction for multiple comparisons were done by Dunnett’s test. *p < 0.05 (against CpG+IL-21 or IgG+IgM+IL-21 as control conditions).

It was then tested whether CQ inhibits regulatory functions of B-cells. Therefore, IL-10 production as wells as GrB synthesis of B-cells was assessed. IL-10^pos^ Breg were induced by CpG, Poly-S or anti-IgG/IgM stimulation. As expected, CpG and Poly-S were potent inducers of IL-10^pos^ Breg whereas the fraction of IL-10^pos^ Breg was low upon anti-IgG/IgM stimulation (**Figure 3** and **Figure S2**). CQ lowered the fraction of CpG-induced Breg significantly at a concentration of 0.5 uM (**Figure 3A**). The same concentration had no significant effect on Poly-S induced Breg (**Figure 3B**). However, at a CQ concentration of 10 uM, the fraction of Poly-S induced Breg was significantly reduced (**Figure 3B**). There was no effect on Breg induced by anti-IgG/IgM stimulation. Next, it was investigated whether Breg producing GrB are affected by CQ. GrB^pos^ Breg were potently induced by stimulation with CpG or anti-IgG/IgM in presence of IL-21 (**Figure 4**). However, BCR-stimulation lead to a higher fraction of GrB^pos^ Breg than CpG-stimulation. CQ suppressed CpG-induced GrB^pos^ Breg whereas inhibition of anti-IgG/IgM-induced GrB**^pos^** Breg was much less effective (**Figures 4** and **S1**).

### Effector B-Cell Functions Such as Plasma Blast Formation and IgG Secretion Are Suppressed by CQ

**Figure 5:**
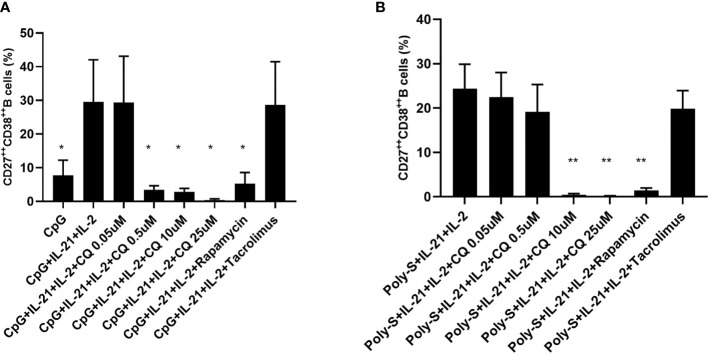
Effect of CQ on plasmablast formation depending on the stimulus. Purified B cells were stimulated for 6 days and were monitored for differentiation into plasma cells by flow cytometry. **(A)** Impact of CQ on plasma cells upon CpG plus IL-21 and IL-2 stimulation. **(B)** Impact of CQ on plasma cells upon IgG+IgM plus IL-21 and IL-2 stimulation. P-values were calculated by repeated-measures ANOVA and correction for multiple comparisons were done by Dunnett’s test. *p < 0.05, **p < 0.001 (against CpG+IL-2+IL-21 or Poly-S+IL-2+IL-21 as control conditions).

**Figure 6:**
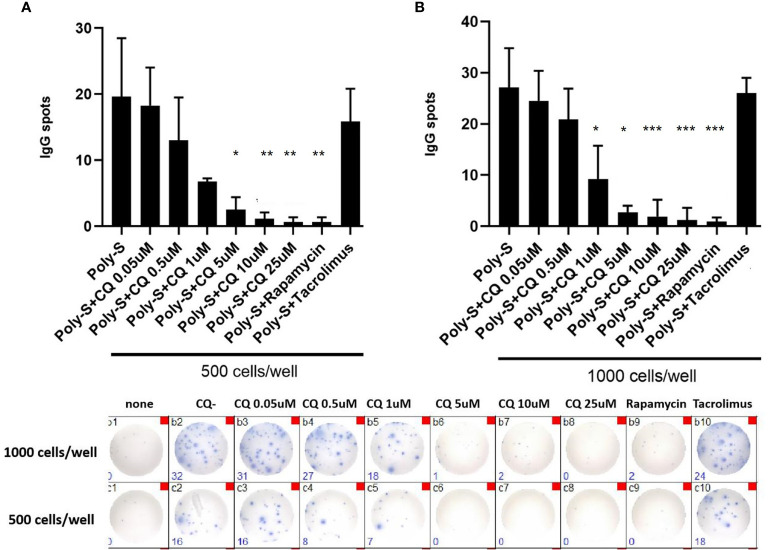
Chloroquine suppresses antibody-synthesis by plasma cells. To assess if CQ has impact on antibody-secretion by B-cells, a human IgG-specific ELISpot analysis was performed. Isolated CD19^+^B cells were initially seeded at 5×10^4^ cells/well under Poly-S stimulation in presence of CQ at different concentrations for four days. Then, cells were harvested and transferred to ELISpot plates at a density of 500 cells/well or 1000 cells/well to be cultured for another 24 hours. **(A, B)** Impact of CQ on IgG-secretion of plasma cells using purified B cells. P-values were calculated by repeated-measures ANOVA and correction for multiple comparisons were done by Dunnett’s test. *p < 0.05, **p < 0.001, ***p < 0.0001 (against Poly-S as control condition).

**Figure 7:**
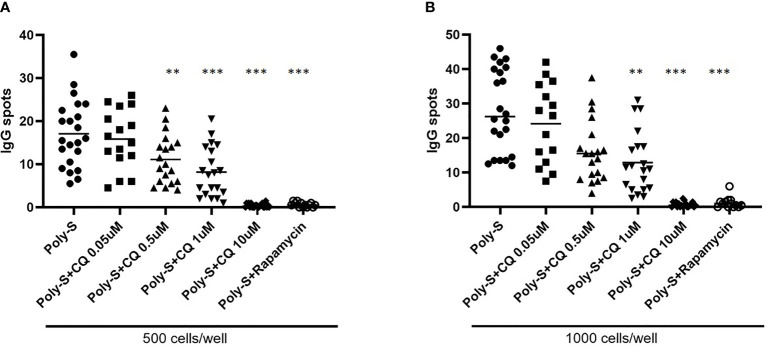
Effect of CQ on IgG-secretion of plasma cells derived from renal transplant patients. To further investigate the effect of CQ on B-cells from renal transplant patients, 22 patients were enrolled. Isolated CD19**^+^** B cells derived from renal transplant patients were seeded at a concentration of 5 × 10^4^ cells/well under Poly-S stimulation in presence of CQ for 4 days. B cells were then harvested and transferred to ELISpot plates at a density of 500 or 1000 cells/well for further 24 hours. **(A)** Impact of CQ on IgG-secretion of plasma cells at a density of 500 cells/well. **(B)** Impact of CQ on IgG-secretion of plasma cells at a density of 1000 cells/well. P-values were calculated by repeated-measures ANOVA and correction for multiple comparisons were done by Dunnett’s test. **p < 0.001, ***p < 0.0001 (against Poly-S as control condition).

Furthermore, it was investigated to which extend CQ has impact on effector B-cell function. Specifically, plasma blast formation and IgG secretion was studied. Plasmablast formation was induced by either stimulation with CpG or Poly-S in presence of IL-21 and IL-2 for six days. The fraction of plasmablasts defined as CD19**^+^** CD27**^++^** CD38**^++^** was then determined by flow cytometry (**Figure S3**). CQ inhibited plasmablast formation efficiently at low concentrations upon TLR9 stimulation with CpG being as potent as rapamycin (**Figure 5A**). CQ was slightly less suppressive upon TLR7-stimulation with Poly-S and higher concentrations were necessary (**Figure 5B**). In the same conditions, rapamycin suppressed plasmablast formation efficiently. Subsequently, it was studied if IgG secretion can be inhibited by CQ. Purified B-cells were stimulated for four days with Poly-S in presence of CQ, tacrolimus or rapamycin. Then, B-cells were transferred to EliSpot plates and SFU were determined after 24 hours. CQ significantly lowered the number of SFU already at a concentration of 1 uM and was equally potent to rapamycin at higher concentrations (**Figure 6**). Tacrolimus did not influence IgG secretion. To assess if CQ also shows significant effects under diseased conditions, B-cells derived from patients after renal transplantation were assayed. CQ inhibited IgG secretion to a similar extend as seen in healthy controls (**Figure 7**).

## Discussion

CQ is used as treatment in autoimmune diseases such as SLE and RA (1, 2). The exact mechanisms of action have not been deciphered completely and the specific effect on B-cells has been investigated scarcely. Thus, this study aimed to assess the impact of CQ on regulatory and effector B-cells. Furthermore, it was tested whether CQ suppresses B-cells to the same extent under diseased conditions. It was found that CQ potently suppressed B-cell proliferation; moreover CQ had a differential impact on regulatory B-cells with a more substantial inhibition of IL-10^pos^ Breg whereas GrB^pos^ Breg were less susceptible to suppression by CQ. CQ inhibited effector B-cell functions such as plasmablast formation and antibody production not only in healthy controls but also in renal transplant patients.

It has been demonstrated previously that CQ exerts its effects on mononuclear cells by different mechanisms of action. CQ antagonizes the TLR-mediated stimulation of mononuclear cells; elevation of intra-lysosomal pH preventing acidification necessary for activation, binding of nucleic acid ligands and interference with downstream signaling seem key mechanisms (3). The impact of CQ on TLR9-mediated effector functions of B-cells was studied by Torigoe et al. (12). CQ blocked the maturation of B-cells into plasma cells, IgG secretion and TNFα production (12). Likewise, in a murine lupus model, CQ blocked TLR9-mediated proliferation of autoantigen-specific B-cells (13). There is fewer data on antagonism of TLR7/8 mediated mononuclear cells. Resiquimod-induced NK cell activation was dampened by CQ in an *in vitro* model (14). Our study confirms the existing data on suppression of TLR9-stimulated effector B-cells by CQ. CQ suppressed TLR9-induced B-cell proliferation and plasmablast formation. Furthermore, our study extends the body of evidence further and provides novel, robust data on CQ-mediated suppression of TLR7/8-stimulated effector B-cells. CQ suppressed resiquimod-induced B-cell maturation into plasmablasts and IgG production suggesting a wider range of inhibitory capacity. TLR7/8 and TLR9 also recognize endogenous ligands known as damage associated molecular patterns (DAMP) which are released upon tissue damage (9–11, 15, 16). In the setting of renal transplantation, TLR7 also recognizes host-derived miRNA; overexpression of specific types within the renal allograft have been associated with reperfusion injury, acute and chronic rejection (17–19). TLR9 signaling is induced by intra-graft release of high mobility group boxed protein-1 (HMGB-1) and mitochondrial DNA (9). Therefore, TLR7/8 and TLR9 induced B-cell activation may contribute to sensitization of B-cells in renal transplant patients. Indeed, despite broad immunosuppressive therapy, allograft rejection occurs in renal transplant patients and especially antibody-mediated rejection is problematic leading to loss of graft function (20). B-cell directed therapy is one of the therapeutic measures that can be considered but comes at the expense of increased risk of infection (21). Thus, the effect of CQ on TLR-stimulated B-cells derived from renal transplant patients was assessed and a profound suppressive effect on effector B-cells was demonstrated. As CQ suppressed maturation and subsequent IgG secretion of B-cells derived from renal transplant patients *in vitro*, CQ could have the potential to further optimize prophylaxis against antibody-mediated rejection (ABMR). This is also interesting as CQ does not increase the risk for infection (4). Still, CQ acts right in the end of a complex cascade leading to formation and maturation of donor-specific B-cells (22). As has been demonstrated earlier, there are several other factors driving maturation of donor-specific B-cells such as the B-cell activating factor (BAFF), IL-6 and interactions with other immune cells (22, 23). It might be more beneficial to interfere very early with this complex cascade and targeting effector B-cells during the already initiated maturation process could be too late.

To which extend Breg are influenced by CQ has not been studied in detail in human B-cells. In our study, we investigated two different subsets of Breg. IL-10^pos^ Breg are pivotal to maintain immunological tolerance and functional or numerical deficiency promotes unwanted inflammation (24–27). IL-10^pos^ Breg promote the development of regulatory T-cells (Treg), directly suppress pro-inflammatory T-cells *via* IL-10 as well as *via* contact-dependent mechanisms and inhibit maturation of antigen-presenting cells (28). Dominance of IL-10^pos^ Breg over effector B-cells has been associated with a more favorable outcome after renal transplantation (29, 30); likewise, patients with antibody-mediated rejection showed a profound deficiency of circulating IL-10^pos^ Breg (31, 32). GrB^pos^ Breg have been studied less extensively but the current evidence suggests a similar role in restraining pro-inflammatory immune responses (33–35). Circulating GrB^pos^ Breg are expanded in renal transplant patients with operational tolerance and during rejection, numbers seem diminished (36). It has been hypothesized that local GrB release by Breg without concomitant expression of perforin may lead to degradation of the T-cell receptor (TCR) of neighboring T-cells (37). We could show that CQ interferes with CpG-induced maturation of Breg, no matter if IL-10 or GrB producing. Likewise, TLR7/8-induced maturation of IL-10^pos^ Breg was hampered. This is in line with data from Miles et al. showing that murine B-cells secret less IL-10 in presence of CQ (38). In another study by Cepika et al. SLE patients were followed before and after beginning of CQ therapy. After initiation of CQ therapy, IL-10 serum levels decreased and IL-10 secretion of total mononuclear cells was diminished upon CpG-stimulation (39).

In contrast, anti-BCR-mediated induction of GrB^pos^ Breg was much less susceptible to CQ treatment consistent with the current concept of CQ-mediated mechanism of action. Rabani et al. studied GrB^pos^ Breg in a cohort of SLE patients (34). In these patients, the fraction of GrB^pos^ Breg was reduced in comparison to healthy controls. However, patients receiving CQ showed a similar fraction of GrB^pos^ Breg as compared to patients who were not treated with CQ.

Thus, Breg show a differential response to CQ with IL-10^pos^ Breg being very susceptible to CQ-mediated suppression whereas GrB^pos^ Breg are nearly unaffected. If CQ would selectively affect Breg sparing effector B-cells, this would have a detrimental effect in B-cell mediated diseases. However, as effector B-cells are also inhibited by CQ, the whole B-cell compartment most probably shifts towards a less pro-inflammatory polarization consistent with the observed clinical efficacy of CQ in B-cell mediated autoimmune diseases.

In summary, we demonstrated that CQ not only suppresses CpG-induced effector B-cell maturation but also resiquimod i.e. TLR7/8-mediated effector B-cell function. Furthermore, this effect was not limited to B-cells from healthy controls but was also reproducible in B-cells from patients after renal transplantation. In addition, we provided novel data that CQ differentially impacts subsets of regulatory B-cells extending the knowledge on the biology and role of these regulatory subsets. These novel insights may allow to further investigate CQ as additional treatment for other patient cohorts.

## Data Availability Statement

The raw data supporting the conclusions of this article will be made available by the authors, without undue reservation.

## Ethics Statement

The studies involving human participants were reviewed and approved by the local institutional review board, Ethik-Kommission Medizinische Fakultät Universität Duisburg-Essen. The patients/participants provided their written informed consent to participate in this study.

## Author Contributions

XM designed the study, performed the experiments, performed the data analyses, and wrote the manuscript. YD, SX, SD, ML, AK, and OW designed the study and critically edited the manuscript. BW designed the study, supervised the study, performed data analyses, and wrote the manuscript. All authors contributed to the article and approved the submitted version.

## Funding

BW was funded by the Dr. Werner Jackstädt-Stiftung, OW received funding from the Rudolf Ackermann-Stiftung. We acknowledge support by the Open Access Publication Fund of the University of Duisburg-Essen.

## Conflict of Interest

The authors declare that the research was conducted in the absence of any commercial or financial relationships that could be construed as a potential conflict of interest.

## Publisher’s Note

All claims expressed in this article are solely those of the authors and do not necessarily represent those of their affiliated organizations, or those of the publisher, the editors and the reviewers. Any product that may be evaluated in this article, or claim that may be made by its manufacturer, is not guaranteed or endorsed by the publisher.

## Supplementary Material

Supplementary Figure 1 Effect of CQ on GrB producing regulatory B-cells: Representative flow cytometric data. Plots are gated on vital B-cells.

Supplementary Figure 2 Effect of CQ on IL-10 producing regulatory B-cells: Representative flow cytometric data. Plots are gated on vital B-cells.

Supplementary Figure 3 Effect of CQ on plasma blast formation of B-cells: Representative flow cytometric data. Plots are gated on vital B-cells and plasma blasts were defined as CD27^++^CD38^++^.
