# Supplementary material for: Chloroquine Suppresses Effector B-Cell Functions and Has Differential Impact on Regulatory B-Cell Subsets
Source: Front Immunol. 2022 Feb 8;13:818704. doi: 10.3389/fimmu.2022.818704 (PMC8860819; doi:10.3389/fimmu.2022.818704)
Supplement: Supplementary Figure 1 — Effect of CQ on GrB producing regulatory B-cells: Representative flow cytometric data. Plots are gated on vital B-cells. [file Image_1.pdf]

S1

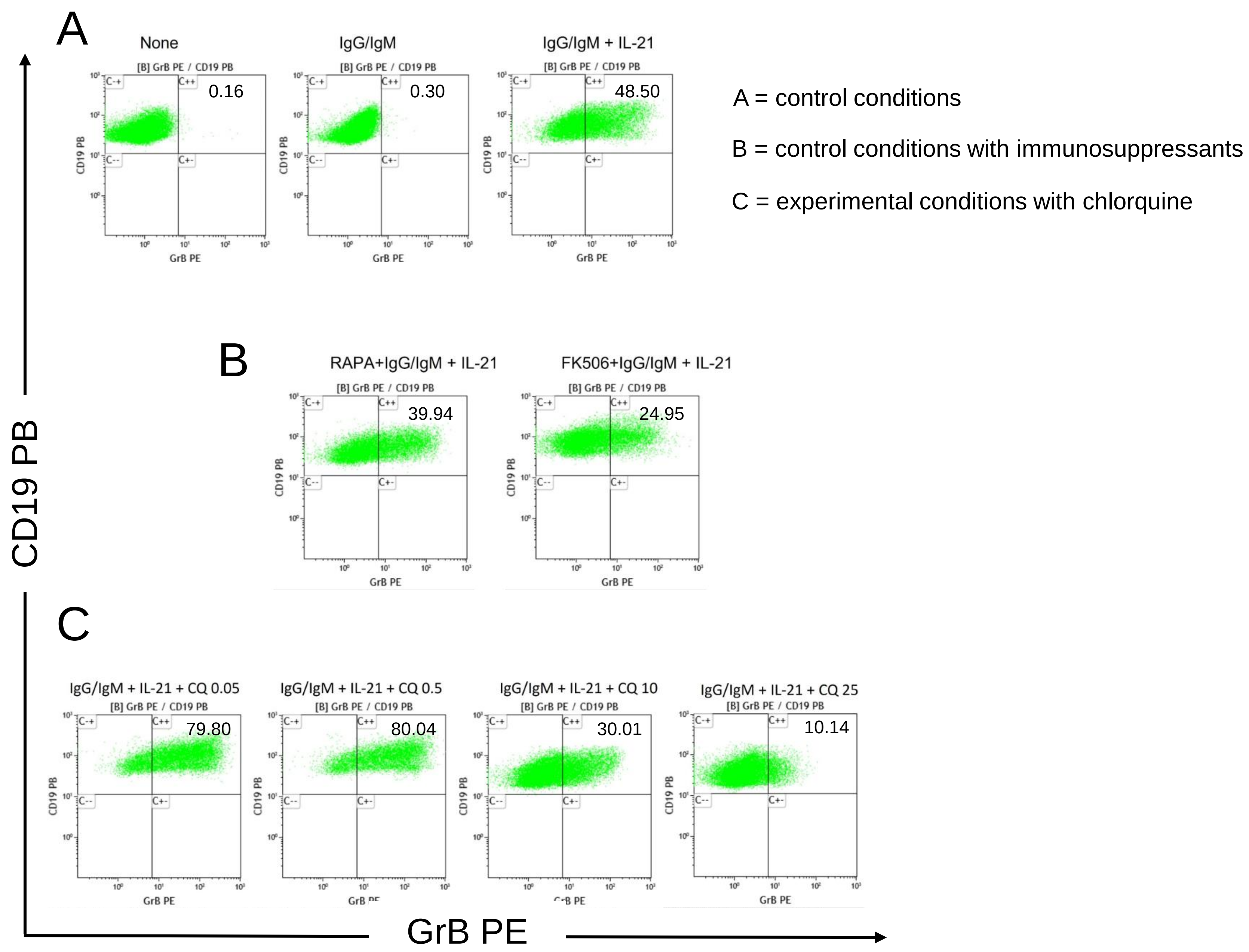

Figure S1. Effect of CQ on GrB producing regulatory B-cells: Representative flow cytometric data.

S2

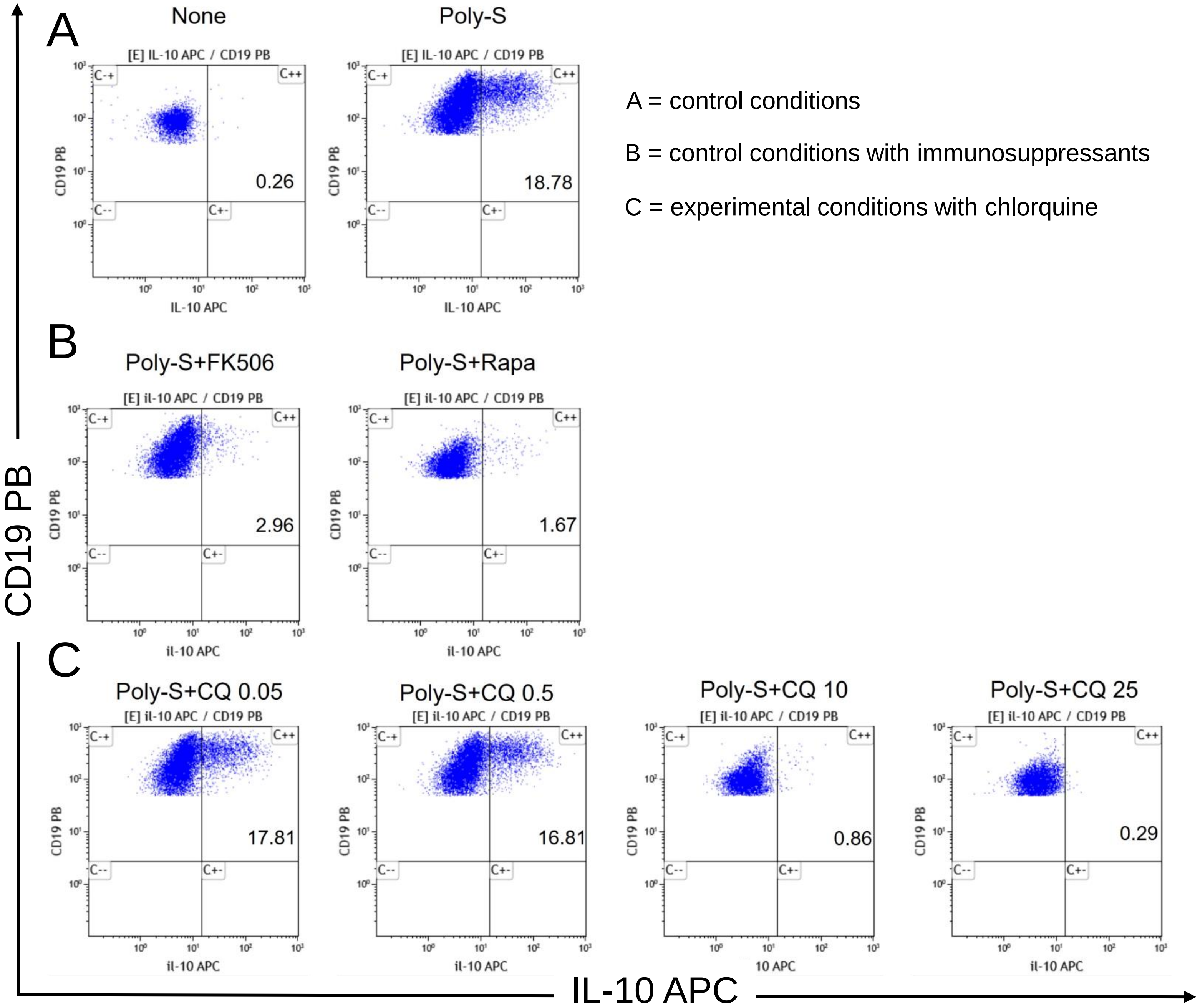

Figure S2. Effect of CQ on IL-10 producing regulatory B-cells: Representative flow cytometric data.

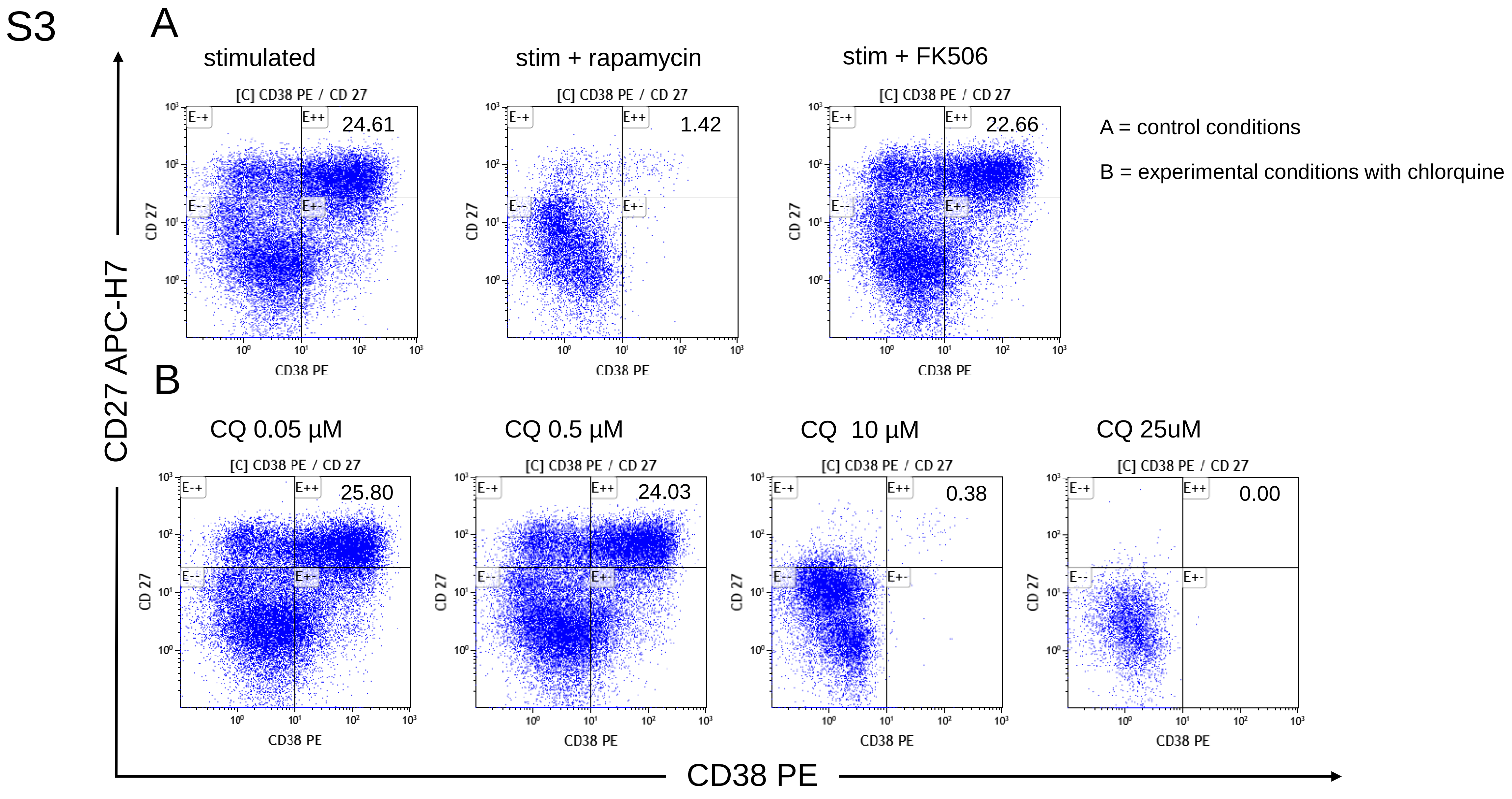

Figure S3. Effect of CQ on plasma blast formation of B-cells: Representative flow cytometric data.
